# A multicentre study comparing post-mortem SARS-CoV-2 antibody testing in Cape Town mortuaries

**DOI:** 10.4102/sajid.v40i1.683

**Published:** 2025-03-31

**Authors:** Tayna Carlisle, Yuvika Vandayar, Laura Taylor, Itumeleng Molefe, Lorna J. Martin, Candice Wilscott-Davids, Janette Verster, Christoffel Opperman, Laura J. Heathfield

**Affiliations:** 1 Division of Forensic Medicine and Toxicology, Department of Pathology, Faculty of Health Sciences, University of Cape Town, Cape Town, South Africa; 2 Division of Forensic Medicine, Department of Pathology, Faculty of Medicine and Health Sciences, Stellenbosch University, Cape Town, South Africa; 3 National Health Laboratory Service, Green Point Tuberculosis Laboratory, Cape Town, South Africa; 4 South African Medical Research Council (SAMRC) Centre for Tuberculosis Research, Division of Molecular Biology and Human Genetics, Stellenbosch University, Cape Town, South Africa; 5 Division of Medical Microbiology, Department of Pathology, Faculty of Health Sciences, University of Cape Town, Cape Town, South Africa

**Keywords:** antibody screening, COVID-19, SARS-CoV-2, serology, post-mortem, lateral flow

## Abstract

**Background:**

Coronavirus disease 2019 (COVID-19) was recognised as a global pandemic in 2019, yet the exact number of infections is still unclear. In addition, there is limited research on post-mortem antibody testing.

**Objectives:**

This study sought to evaluate the use of the SureScreen COVID-19 immunoglobulin (Ig) G and IgM Rapid Test Cassette in deceased individuals by comparing it to the gold-standard antibody tests in South Africa, and to identify the most appropriate antibody testing method for post-mortem samples.

**Method:**

Between May 2021 and February 2023, fifty cases, with suspected COVID-19 infection during their lifetime, were recruited from Tygerberg Mortuary and Salt River Mortuary, after obtaining informed consent from their next-of-kin. Severe acute respiratory syndrome coronavirus 2 (SARS-CoV-2) infection was confirmed through antemortem positive COVID-19 polymerase chain reaction (PCR) (PCP) tests in 39 participants. Blood samples were collected during autopsies in serum separator tubes, which yielded better separation when centrifuged immediately after collection. The SureScreen test was performed alongside Roche Diagnostics Elecsys Anti-SARS-CoV-2 and Abbott Architect SARS-CoV-2 IgG assays.

**Results:**

Among the confirmed PCP cases, Elecsys demonstrated the highest sensitivity (97.1%) followed by SureScreen IgG (82.1%). In a logistic regression analysis, PCP confirmation was significantly associated with the SureScreen IgG results (*p* < 0.05).

**Conclusion:**

Overall, Roche’s Elecsys had the highest yield of positive results on our cohort of post-mortem serum samples, followed by SureScreen, and finally, Abbott’s Architect assay.

**Contribution:**

These results suggest that the SureScreen test has potential as a screening tool in mortuary settings, with Roche’s Elecsys assay recommended for diagnostic confirmation.

## Introduction

The coronavirus disease 2019 (COVID-19) pandemic, caused by the severe acute respiratory syndrome coronavirus 2 (SARS-CoV-2) virus, led to more than 7 million deaths globally.^1^ Once a person is infected, the production of immunoglobulin (Ig) M, IgG3, IgG1 and IgA antibodies to the viral envelope (E), nucleocapsid (N) and spike (S) proteins are initiated.^2,3^ After infection, IgM and IgA appear around five days, followed by IgG at approximately 14 days.^2,4,5,6^ Studies suggest that IgG can be detected up to 12 months post SARS-CoV-2 infection.^7,8^

The Abbott Architect SARS-CoV-2 IgG Assay (hereinafter referred to as Architect) and the Roche Elecsys Anti-SARS-CoV-2 (hereinafter referred to as Elecsys) are two assays used for SARS-CoV-2 antibody testing. The Architect assay detects IgG antibodies that are reactive against the N protein of SARS-CoV-2 in human serum and plasma,^9^ and the Elecsys assay detects all antibodies (including IgG) to SARS-CoV-2 N protein in human serum and plasma.^10^ Severe acute respiratory syndrome coronavirus 2 antibody testing is also available as a lateral flow immunoassay (LFI), which can be performed either by the patient or a healthcare professional in many settings.^11^ The SureScreen COVID-19 IgG and IgM Rapid Test Cassette (SureScreen rapid test) is a membrane-based LFIs and was developed for the detection of anti-SARS-CoV-2 N protein antibodies – IgG and IgM using whole blood, serum, or plasma.^5^ In clinical settings, SureScreen tests had reported sensitivities of 97.4% (IgG) and 86.8% (IgM), and specificities of 99.3% (IgG) and 98.6% (IgM), whereas the Elecsys and Architect assays had sensitivities of 99.5% and 100%, and specificities of 99.8% and 99.6%, respectively.^12,13,14,15^

In the post-mortem setting, monitoring prior SARS-CoV-2 infections is crucial for enhancing surveillance and public health response. Limited research exists on anti-SARS-CoV-2 antibody testing in deceased individuals, and no study has assessed the use of LFIs to date. Antibody testing in post-mortem samples is also less reliable because of processes such as haemolysis, autolysis, and bacterial contamination.^16,17^ This study aimed to investigate the effectiveness of the SureScreen test in the deceased population by comparing its results to the Elecsys and Architect assays.

## Research methods and design

### Participants and sample collection

Fifty participants were recruited from Tygerberg Mortuary (*n* = 15) and Salt River Mortuary (*n* = 35) in Cape Town over the course of 1 year. Cases were included if the deceased (1) was ≥ 18 years, (2) suspected of having COVID-19 in their lifetime (irrespective of when the infection took place), (3) showed no macroscopic signs of decomposition and (4) if their next-of-kin provided informed consent. Information on possible previous COVID-19 infection was gathered upon admission of the bodies to the mortuaries as part of the routine operational procedure. The procedure is explained to the next-of-kin during consent sessions and confirmed by formal laboratory records. Nineteen participants were female and 31 were male (ages 19–92 years old). Positive COVID-19 polymerase chain reaction (PCP) was confirmed in 39 of the participants.

Two blood samples (each 4 mL) were collected from each participant into serum separator tubes (SSTs) at autopsy. Following autopsy, samples were stored at 4 °C at the mortuary and for no longer than 12 h before transportation to the respective laboratories. Salt River Mortuary case samples were taken to the National Health Laboratory Service (NHLS) at Groote Schuur Hospital and samples from Tygerberg Mortuary were transported to Bio Analytical Research Corporation South Africa (BARC). Samples were centrifuged at 4000 *× g* for 10 min and stored at 2–8 °C until testing at each site.

### Antibody testing

Serum (10 µL) was tested using the SureScreen test according to the manufacturer’s protocol. After 10 min the results were interpreted. Any shade of colour in the IgG and/or IgM test line region(s) was considered positive, provided the control worked; the read-outs were performed by the examiners. The remaining serum from this first vial was analysed using Elecsys according to the manufacturer’s protocol.^10^ Cases 9–12 were analysed using Elecsys S Total Antibody test while the others were tested using the Elecsys N total antibody test. At the time of this study, the Elecsys assay was routinely used at the NHLS for SARS-CoV-2 antibody testing in the public healthcare sector of South Africa. However, Groote Schuur NHLS suspended their offering of the Elecsys assay towards the end of this study because of low demand, thus the last four cases do not have Elecsys data and were excluded from statistical analyses.

Serum from the second vial of blood was tested using the Architect assay, which at the time of the study, was only available in the private healthcare sector in South Africa. Testing was performed using the manufacturer’s protocol with no deviations.^9^

The regulatory requirements for rapid test kits are determined based on the existing regulatory framework for medical devices and in vitro diagnostics (IVDs) implemented in 2016, which are in line with the World Health Organization Global Model Regulatory Framework for Medical Devices.^18^ All the three antibody tests used in this study have been validated in South Africa and approved by SAHPRA under Section 21 authorisation for unregistered medical devices and IVDs.^14,15,18,19,20^

### Data analysis

The data were summarised using descriptive statistics. The number of days between PCP, death, autopsy, and antibody testing were calculated for each case, as well as the respective medians. For the cases without PCP confirmation, the month and year as reported by next-of-kin (where available) were used in the data analyses.

Pairwise comparisons of results from the three antibody tests were performed. These included Fisher’s exact tests and percent overall agreement (POA) (Online Appendix 1: Equation 1-A1). The sensitivities of each antibody test were calculated using the cases with PCP confirmation (*n* = 39; Online Appendix 1: Equation 2-A1). To facilitate comparison to published clinical data, the sensitivities were calculated using the following intervals between PCP and testing: 0–6 days, 7–13 days, > 14 days.^11^ However, because of the intervals between PCP and testing being substantially longer in this study, sensitivities were also calculated using the following intervals: 0–5 days, 6–50 days, 51–200 days, 201–600 days, and 600+ days.

Specificity and accuracy were not determined because there were no cases in the study cohort where it was confirmed that the individual had never had COVID-19 during their lifetime (i.e., no true negative cases).

A logistic regression analysis was performed to elucidate which variables significantly influenced the test results. This included PCP confirmation and the intervals between PCP, death, autopsy, and post-mortem testing. Vaccination status was not included in the logistic regression analysis as the information could not be verified. Two cases lacked dates for COVID-19 infection and were therefore excluded from the model. Collinearity among the variables was assessed using a Pearson correlation test. The number of days between PCP and death, PCP and autopsy, and PCP and testing were collinear to each other (correlation > 0.8), thus only one was included in the logistic regression. All statistical analyses were performed using the R statistical analysis application and an alpha < 0.05 was considered statistically significant.

### Ethical considerations

Ethical clearance to conduct this study was obtained from the University of Cape Town Faculty of Health Sciences Human Research Ethics Committee (reference no.: HREC: 637/2020) and the Stellenbosch University Health Research Ethics Committee (reference no.: M21/03/002_RECIP_UCT_637/2020_COVID-19) for each recruitment site. Approval was also obtained from the South African Health Products Regulation Association (SAHPRA) (reference no.: MD20201201) and National Health Research Database (NHRD) (reference no.: WC_202111_025).

## Results

### Overview

The results of each test, along with case demographics, PCP confirmation, vaccination status, and the time intervals between infection, death, autopsy, and antibody testing, are tabulated in Table 1^21^ with each case detailed in Online Appendix 1: Table 1-A1. Twelve participants received vaccinations against SARS-CoV-2, 16 were unvaccinated and the remaining 22 were unknown. The results of each antibody test are presented in the following sub-sections.

**TABLE 1:** A summary of the participant variables and the three COVID-19 antibody tests.

Test	Total number			Cases with PCP confirmation			Cases vaccinated			Days between											
										PCP and deat		PCP and autopsy		Death and autopsy		Death and testing		PCP and testing		Autopsy and testing	
	*n*	*N*	%	*n*	*N*	%	*n*	*N*	%	Median	Range	Median	Range	Median	Range	Median	Range	Median	Range	Median	Range
SS IgM +	8	50	16.0	7	8	87.5	3	8	37.5	7.5	0–702	13.5	3–706	4.0	3–8	7.5	4–20	24.0	7–709	4.0	0–14
SS IgM −	42	50	84.0	32	42	76.2	9	42	21.4	227.0	0–848	235.0	2–850	3.0	1–14	8.0	1–31	240.5	6–850	4.0	0–29
SS IgG +	38	50	76.0	32	38	84.2	12	38	31.6	200.0	0–848	202.5	2–850	4.0	1–11	7.0	1–31	206.0	6–850	4.0	0–29
SS IgG −	12	50	24.0	7	12	58.3	0	12	0.0	81.0	1–625	82.0	5–630	3.5	1–14	10.0	2–19	89.0	11–643	6.5	0–13
Elecsys +	43	46	93.5	34	43	79.1	8	43	18.6	136.0	0–848	140.0	2–850	4.0	1–14	13.0	4–32	143.0	7–864	8.0	0–26
Elecsys −	3	46	6.5	1	3	33.3	1	3	33.3	7.0	6–347	14.0	13–353	6.0	6–8	20.0	14–23	26.0	21–370	12.0	8–17
Architect +	17	50	34.0	12	17	70.6	2	17	11.8	110.5	0–848	118.0	2–850	3.0	1–11	8.0	2–32	119.5	7–865	6.0	1–30
Architect −	33	50	66.0	27	33	81.8	10	33	30.3	238.0	0–812	246.5	3–816	4.0	1–14	9.0	3–21	250.0	6–805	6.0	0–18

*Source:* Adapted from Carlisle T. Comparison of SARS-CoV-2 rapid tests and formal serological testing on deceased persons in Cape Town metro [minor research dissertation]. Cape Town: University of Cape Town; 2022 [cited n.d.]. Available from: https://open.uct.ac.za/server/api/core/bitstreams/c2518868-20eb-4917-a756-48fbcc22d9fc/content

PCP, positive COVID-19 polymerase chain reaction; IgM, immunoglobulin M; IgG, immunoglobulin G.

#### SureScreen test results

Of the 50 cases, 38 (76.0%) were seropositive based on SureScreen rapid assays, with positive results indicated by the IgG marker (*n* = 38) and IgM marker (*n* = 8; Table 1). Of the 42 SureScreen IgM negative cases, 71.4% (*n* = 30) were positive for IgG. There were seven instances where IgM and IgG were both negative but COVID-19 infection was confirmed by PCP (cases 4, 20, 25, 28, 32, 33, and 39) (Online Appendix 1: Table 1-A1). In cases 25 and 28, there was only one day between PCP and death, thus limiting the time for seroconversion. However, there were five cases where the interval between PCP and death was zero days (cases 14, 16, 17, 41, 43; see Table 1 and Online Appendix 1: Table 1-A1). Despite this, all these cases had a positive IgG result and two also had a positive IgM result.

Table 1 shows that if the number of days between PCP and death were high (> 600), positive IgG and IgM results were present regardless of whether the case was confirmed to be vaccinated or unknown (cases 10 and 22).

#### Elecsys test results

For the Elecsys’ assay, 93.0% of test results were positive (*n* = 43/46; see Table 1), with this test only providing three negative results (7.0%; cases 8, 9, and 23; see Online Appendix 1: Table 1-A1). Case 23 had PCP and vaccination confirmation, yet still yielded a negative result. There was, however, a longer time interval between autopsy and testing. The four cases analysed by the Elecsys Anti-SARS-CoV-2 S Total Antibody test had a concentration of > 250 µ/mL, indicating a past natural SARS-CoV-2 infection.

#### Architect test results

Positive results were reported in 34.0% of cases tested using the Architect assay (*n* = 17/50; see Table 1). A high proportion of negative results occurred in cases with confirmed PCP (*n* = 27/39). Architect’s negative results also had a high percentage of PCP confirmation (*n* = 27/33; 81.8%) and vaccinated cases (*n* = 10/33; 30.3%).

#### Test comparisons

Table 2 shows the overall sensitivities of the antibody tests for the 39 cases with confirmed PCP. Elecsys demonstrated a high sensitivity at 97.1% with SureScreen IgG at 82.1%. SureScreen IgM had a low sensitivity at 17.9%.

**TABLE 2:** Sensitivity analyses for various tests at different intervals of time between positive COVID-19 polymerase chain reaction and death with intervals outlined by Elecsys’ instructions for use.

Days between PCP and death	Number of cases			SureScreen IgM sensitivity			SureScreen IgG sensitivity			Elecsys sensitivity			Architect sensitivity		
	*n*	*N*	%	*n*	*N*	%	*n*	*N*	%	*n*	*N*	%	*n*	*N*	%
0–6	8	39	20.5	3	8	37.50	6	8	75.0	6	7	85.7	1	8	12.5
7–13	4	39	10.3	2	4	50.00	3	4	75.0	4	4	100.0	3	4	75.0
>14	27	39	69.2	2	27	7.41	23	27	85.2	24	24	100.0	8	27	29.6

**Total**	**39**	**39**	**100.0**	**7**	**39**	**17.90**	**32**	**39**	**82.1**	**34**	**35**	**97.1**	**12**	**39**	**30.8**

PCP, positive COVID-19 polymerase chain reaction test; IgM, immunoglobulin M; IgG, immunoglobulin G.

For SureScreen IgM and Architect, the highest sensitivities were observed in cases with 7–13 days between PCP confirmation and death. SureScreen IgG showed the highest sensitivity when the interval was more than 14 days. Elecsys achieved 100% sensitivity for both 7–13 days and over 14 days intervals (Table 2). The lowest sensitivity was seen in Architect for the interval of 0–6 days, and in SureScreen IgM for intervals exceeding 14 days.

Further analysis of other relevant intervals revealed that the Elecsys assay maintained consistent performance across all ranges, with the lowest sensitivity occurring at 6–50 days (Online Appendix 1: Table 2-A1). Notably, the sensitivity for SureScreen IgM increased from 0% to 25.0% in the 600+ days category. SureScreen IgG performed best in the intervals where SureScreen IgM had 0% sensitivity. Architect displayed low sensitivities across all intervals, with the highest sensitivity at 40.0% and the lowest at 14.3%.

Table 3 presents the percentage agreements between each pair of tests, along with *p*-values to assess significant differences among the results from the three tests. Of all the pairwise comparisons, Elecsys and SureScreen IgG showed the highest agreement at 76.1%, while Elecsys and SureScreen IgM had the lowest agreement at 19.6%. Additionally, the SureScreen IgG results were significantly different from those of the Architect test, with a *p*-value of 0.004.

**TABLE 3:** Summary of percentage overall agreement and *p*-values comparing different assays.

Test type	SS IgM		SS IgG		Elecsys		Architect	
	%	*p*	%	*p*	%	*p*	%	*p*
SS IgM	-	-	-	0.173	-	0.444	-	0.419
SS IgG	40.0	-	-	-	-	0.162	-	0.004
Elecsys	19.6	-	76.1	-	-	-	-	0.542
Architect	66.0	-	58.0	-	41.3	-	-	-

Note: Percentage represents percentage overall agreement.

SS, SureScreen; IgM, immunoglobulin M; IgG, immunoglobulin G.

Cases 47–50 were analysed with the Architect system at Tygerberg NHLS instead of Elecsys at Groote Schuur NHLS because of a lack of demand of the service at the NHLS. The results for the Tygerberg NHLS Architect testing and BARC Architect testing had a 100% agreement.

### Assessment of variables that may affect the tests’ results

A logistic regression showed that only PCP confirmation was significantly associated with the SureScreen IgG results (*p* < 0.05). The remaining time intervals were not significantly associated with the antibody test results for any of the three tests (*p* > 0.05). Furthermore, there was no significant association between the test results and the age or sex of the participants (Online Appendix 1: Table 3-A1).

No significant correlation was observed when the SureScreen and Architect test results were compared against the Elecsys results using logistic regression analysis (Online Appendix 1: Table 4-A1). For clarity, the Elecsys assay was used for comparison purposes as it produced the highest sensitivity.

## Discussion

### SureScreen rapid test for post-mortem samples

As routine COVID-19 antibody testing is not typically conducted on post-mortem samples, this study explored the parameters of such testing and aimed to develop an optimal procedure. It was found that positive COVID-19 antibody results could be detected beyond 2 weeks after death using the Elecsys test, and up to 11 days with the SureScreen and Architect tests (Online Appendix 1: Table 1-A1). This finding is significant in a forensic mortuary setting, where the average time between death and autopsy is typically three days in Western Cape mortuaries.^22^

The SureScreen testing procedure recommends that to avoid haemolysis, the serum or plasma must be separated immediately after sample collection.^14^ These recommendations were echoed by other studies.^23,24^ Because centrifuges are not commonly available at forensic mortuaries, serum separation could not be performed immediately. As a result, samples were transported to laboratories for separation. This occurred within 12 h of sample collection, however, it was found that the longer the delay before sample centrifuging, the less likely it was to produce serum. If a sample was submitted to the NHLS or BARC without serum, then testing was not able to be performed. This underscores the advantage of LFIs, as they are suitable for point-of-care testing, in contrast to more formal antibody testing methods.^9,13^

Storage recommendations for serum samples differed among the tests. According to SureScreen guidelines, serum samples can be stored at 2 °C – 8 °C for up to 3 days after collection. In contrast, Elecsys and Architect guidelines allow serum to be stored at 2 °C – 8 °C for up to 7 days.^9,10,14^ This study did not impose restrictions on the time between sample collection and testing to ensure a realistic workflow and enhance the practical applicability of the results. For instance, the NHLS performed Elecsys testing only on Mondays, while BARC required sample transport from their Cape Town branch to Johannesburg. Despite these logistical delays, cases with days between autopsy and testing exceeding the manufacturers’ recommendations still produced positive results. The time delays seemed to have no notable association with the antibody test results, as shown in the logistic regression analysis.

### Results of the antibody tests in comparison to existing data

The Elecsys test demonstrated the highest overall sensitivity at 100% for both 7–13 days and over 14 days post-PCP (Table 2). This higher sensitivity may be a result of Elecsys detecting a broader range of COVID-19 antibodies, including IgM, IgG, and IgA^25^ while SureScreen detects only IgM and IgG, and Architect detects specifically IgG. The Elecsys reports do not indicate which specific antibody was detected but provide quantitative results for antibodies generated against the SARS-CoV-2 N protein.

SureScreen IgM’s 100% sensitivity at 7–13 days post-PCP correlates with literature stating that IgM appears approximately five days after infection.^26,27,28^ This also explains the higher sensitivity of SureScreen IgM at 0–6 days post-PCP compared to the IgG tests. However, in this study, SureScreen IgM’s sensitivity increased from 0% for intervals of 51–600 days between PCP and death to 25.0% for cases with over 600 days between PCP and death. This finding contrasts with results from Liu et al. (2019) and Li et al. (2020), who found that IgM was undetectable in patients more than 12 weeks after the initial infection.^26,28^

The findings of this study indicated lower sensitivities for the Architect assay than those reported by the manufacturer.^9^ As the Architect assay tests for IgG, it was hypothesised that its results would align with those of SureScreen IgG. However, the results from these two tests significantly differed from each other despite targeting antibodies to the same *N* protein (*p* = 0.01; see Table 3). The studies reporting sensitivities for the Architect test typically involved hospitalised patients with severe COVID-19, who had very high immune system activity.^9,29^ This study involved a longer time period between PCP and death, which may have contributed to the decreased sensitivity observed.^9^

This introduces another intriguing factor: the duration for which COVID-19 antibodies remain detectable after infection. In this study, the time between PCP and death ranged from 0 to 848 days. A study by De Giorgi et al. (2021) found that, among 116 cases, 91.4% had detectable IgG levels up to 11 months (approximately 330 days) after symptom recovery.^30^ In this study, sensitivities were 91.7% for SureScreen IgG and 100% for Elecsys at 200–600 days between PCP and death, and 87.5% and 100%, respectively, for periods exceeding 600 days (Online Appendix 1: Table 2-A1). While these results are specific to the study population and direct conclusions cannot be generalised, positive COVID-19 antibody test results were observed even 600+ days from PCP until death.

The POA results indicated the highest agreement between Elecsys and SureScreen IgG at 76.1% and the lowest agreement between Elecsys and SureScreen IgM at 19.6%. The POA between Elecsys and Architect was also low, at 41.3% (Table 3). A study by Tan et al. (2021) found that Elecsys slightly outperformed Architect at critical time points of 14 and 21 days. This difference was attributed to Elecsys measuring total antibodies, whereas Architect specifically detects IgG.^31^

Suhandynata et al. (2021) reported that antibody tests detecting only N protein antibodies are unlikely to identify individuals vaccinated against the S protein.^32^ The tests used in this study (SureScreen, Elecsys, and Architect) were designed to detect antibodies in the N protein.^9,14,25^ However, in four instances a different version of the Elecsys assay was performed that targeted SARS-CoV-2 N antibodies and S antibodies (cases 9, 10, 11 and 12). Only one of these cases (case 10) was confirmed to have been vaccinated against SARS-CoV-2. The change in the test was because of shortage of the reagent used for the original Elecsys testing of other cases. However, the concentration of antibodies detected was high (> 250 µ/mL). Considering this result and that testing is mainly N protein based, while vaccinations in South Africa are S protein based, it is more likely that positive results are from natural infection and not vaccination.^32,33,34,35^ In addition, there was no evidence suggesting that sex or age affected the test results.

### Limitations and future work

A limitation of this study was the inability to include negative controls. During the study period, it was not possible to recruit cases where individuals had never had COVID-19. Even if the deceased had an antemortem record of a negative COVID-19 polymerase chain reaction test, infections post testing may have occurred and not been detected. Specificity is crucial for identifying false positive results and evaluating the suitability of antibody tests. The lack of true negative controls hindered a comprehensive analysis, so the recommendations from this study are based solely on test sensitivity.

## Conclusion

The Roche Elecsys Anti-SARS-CoV-2 had the highest yield of positive results on the post-mortem serum samples in this cohort. The SureScreen IgG and IgM rapid test cassette showed adequate performance for IgG detection. Despite lower sensitivity compared to the Elecsys assay it may have utility where point-of-care testing may be a requirement. Based on this study, the Elecsys assay is recommended as the most suitable for diagnosing past infection compared to SureScreen and Architect. This study further showed that antibody testing results were not impacted by the time intervals since sample collection.

This conclusion is concomitant with the SARS-CoV-2 vaccines that are available in the region. If vaccines continue to include the SARS-CoV-2 S protein, these tests will remain effective for detecting natural infections. However, if vaccines containing the N protein are introduced, it may impact the utility of these tests. Overall, this study provides the first empirical data on the SureScreen IgG and IgM rapid test cassette, Roche Elecsys Anti-SARS-CoV-2 assay, and Abbott Architect SARS-CoV-2 IgG assay for post-mortem samples. These findings can guide future research and contribute to COVID-19 surveillance in mortuary settings through anti-SARS-CoV-2 antibody testing.

## References

[1] World Health Organization. WHO coronavirus (COVID-19) dashboard [homepage on the Internet]. 2024 [cited 2024 Jan 18]. Available from: https://covid19.who.int/

[2] French MA, Moodley Y. The role of SARS-CoV-2 antibodies in COVID-19: Healing in most, harm at times. Respirology. 2020;25(7):680–682. 10.1111/resp.13852 32436320 PMC7280731

[3] Sun B, Feng Y, Mo X, et al. Kinetics of SARS-CoV-2 specific IgM and IgG responses in COVID-19 patients. Emerg Microbes Infect. 2020;9(1):940–948. 10.1080/22221751.2020.1762515 32357808 PMC7273175

[4] Tollånes MC, Bakken Kran AM, Abildsnes E, Jenum PA, Breivik AC, Sandberg S. Evaluation of eleven rapid tests for detection of antibodies against SARS-CoV-2. Clin Chem Lab Med. 2020;58(9):1595–1600. 10.1515/cclm-2020-0628 32598303

[5] Nakano Y, Kurano M, Morita Y, et al. Time course of the sensitivity and specificity of anti-SARS-CoV-2 IgM and IgG antibodies for symptomatic COVID-19 in Japan. Sci Rep. 2021;11(1):1–10. 10.1038/s41598-021-82428-5 33531605 PMC7854735

[6] Fernández-Rodríguez A, Casas I, Culebras E, Morilla E, Cohen MC, Alberola J. COVID-19 and post-mortem microbiological studies. Span J Legal Med. 2020;46(3):127–138. 10.1016/j.remle.2020.05.007

[7] Xiao K, Yang H, Liu B, et al. Antibodies can last for more than 1 year after SARS-CoV-2 infection: A follow-up study from survivors of COVID-19. Front Med (Lausanne). 2021;8:1–10. 10.3389/fmed.2021.684864 PMC8322582 34336891

[8] Chansaenroj J, Yorsaeng R, Puenpa J, et al. Long-term persistence of severe acute respiratory syndrome coronavirus 2 (SARS-CoV-2) spike protein-specific and neutralizing antibodies in recovered COVID-19 patients. PLoS One. 2022;17(4):1–16. 10.1371/journal.pone.0267102 PMC9022880 35446889

[9] Abbott Laboratories. SARS-CoV-2 IgG for use with architect [homepage on the Internet]. 2021 [cited 2022 May 30]. Available from: https://www.fda.gov/media/137383/download

[10] Roche Diagnostics. Elecsys^®^ anti-SARS-CoV-2 S [homepage on the Internet]. 2022 [cited 2022 Jul 28]. Available from: https://diagnostics.roche.com/global/en/products/params/elecsys-anti-sars-cov-2-s.html#productSpecs

[11] Koczula KM, Gallotta A. Lateral flow assays. Essays Biochem. 2016;60(1):111–120. 10.1042/EBC20150012 27365041 PMC4986465

[12] Muench P, Jochum S, Wenderoth V, et al. Development and validation of the Elecsys anti-SARS-CoV-2 immunoassay as a highly specific tool for determining past exposure to SARS-CoV-2. J Clin Microbiol. 2020;58(10):e01694-20. 10.1128/JCM.01694-20 32747400 PMC7512151

[13] Roche Diagnostics. Elecsys^®^ anti-SARS-CoV-2 immunoassay for the qualitative detection of antibodies against SARS-CoV-2 [homepage on the Internet]. 2020 [cited 2022 May 30]. Available from: https://diagnostics.roche.com/us/en/products/lab/elecsys-anti-sars-cov-2-cps-000273.html

[14] SureScreen Diagnostics. COVID-19 coronavirus rapid test cassette. 2020 [cited 2020 Aug 12]. Available from: https://www.surescreen.com/products/covid-19-coronavirus-rapid-test-cassette

[15] Jugwanth S, Gededzha MP, Mampeule N, et al. Performance of the Abbott SARS-CoV-2 IgG serological assay in South African 2 patients. PLoS One. 2022;17(2):e0262442. 10.1371/journal.pone.0262442 35120133 PMC8815965

[16] Wilkemeyer I, Pruss A, Kalus U, Schroeter J. Comparative infectious serology testing of pre- and postmortem blood samples from cornea donors. Cell Tissue Bank. 2012;13:447–452. 10.1007/s10561-012-9326-0 22802139

[17] Victer TNdF, Dos Santos CSR, Báo SN, Sampaio TL. Deceased tissue donor serology and molecular testing for HIV, hepatitis B and hepatitis C viruses: A lack of cadaveric validated tests. Cell Tissue Bank. 2016;17:543–553. 10.1007/s10561-016-9564-7 27329292

[18] South African Health Products Regulatory Authority. SAHPRA approves SARS-CoV-2 serology test kits [homepage on the Internet]. 2020 [cited 2023 Jun 01]. Available from: https://www.sahpra.org.za/wp-content/uploads/2020/08/Media-release-SAHPRA-approves-SARS-COV-2-serology-test-kits-.pdf

[19] SAHPRA. SAHPRA registered health products [homepage on the Internet]. 2022 [cited 2022 Jul 24]. Available from: https://www.sahpra.org.za/registered-health-products/

[20] Grove JS, Mayne ES, Burgers WA, et al. Validation of Roche immunoassay for severe acute respiratory coronavirus 2 in South Africa. S Afr J Infect Dis. 2021;36(1):1–6. 10.4102/sajid.v36i1.286 PMC11457558 39376942

[21] Carlisle T. Comparison of SARS-CoV-2 rapid tests and formal serological testing on deceased persons in Cape Town metro [minor research dissertation]. Cape Town: University of Cape Town; 2022 [cited n.d.]. Available from: https://open.uct.ac.za/server/api/core/bitstreams/c2518868-20eb-4917-a756-48fbcc22d9fc/content

[22] Reid KM, Martin LJ, Heathfield LJ. Bodies without names: A retrospective review of unidentified decedents at Salt River Mortuary, Cape Town, South Africa, 2010–2017. S Afr Med J. 2020;110(3):223–228. 10.7196/SAMJ.2020.v110i3.14192 32657700

[23] Edler C, Wulff B, Schröder AS, et al. A prospective time-course study on serological testing for human immunodeficiency virus, hepatitis B virus and hepatitis C virus with blood samples taken up to 48 h after death. J Med Microbiol. 2011;60(7):920–926. 10.1099/jmm.0.027763-0 21436373

[24] Kalus U, Wilkemeyer I, Caspari G, et al. Validation of the serological testing for anti-HIV-1/2, anti-HCV, HBsAg, and anti-HBc from post-mortem blood on the siemens-BEP-III automatic system. Transfus Med Hemotherapy. 2011;38(6):365–372. 10.1159/000334481 PMC3267999 22403520

[25] Roche Diagnostics. Elecsys anti-SARS-CoV-2 S [homepage on the Internet]. 2021 [cited 2021 Aug 11]. Available from: https://www.fda.gov/media/144037/download

[26] Liu R, Liu X, Yuan L, et al. Analysis of adjunctive serological detection to nucleic acid test for severe acute respiratory syndrome coronavirus 2 (SARS-CoV-2) infection diagnosis. Int Immunopharmacol. 2020;86:106746. 10.1016/j.intimp.2020.106746 32619956 PMC7318959

[27] Ripperger TJ, Uhrlaub JL, Watanabe M, et al. Orthogonal SARS-CoV-2 serological assays enable surveillance of low-prevalence communities and reveal durable humoral immunity. Immunity. 2020;53(5):925–933.e4. 10.1016/j.immuni.2020.10.004 33129373 PMC7554472

[28] Li Z, Yi Y, Luo X, et al. Development and clinical application of a rapid IgM-IgG combined antibody test for SARS-CoV-2 infection diagnosis. J Med Virol. 2020;92(9):1518–1524. 10.1002/jmv.25727 32104917 PMC7228300

[29] Johnston S, McKay P, Kebadze T, et al. Evaluation of the Abbott architect, Roche Elecsys and virtus S1 SARS-CoV-2 antibody tests in community-managed COVID-19 cases. medRxiv. 2020;10(7):20220509. 10.1101/2020.10.27.20220509

[30] De Giorgi V, West KA, Henning AN, et al. Naturally acquired SARS-CoV-2 immunity persists for up to 11 months following infection. J Infect Dis. 2021;224(8):1294–1304. 10.1093/infdis/jiab295 34089610 PMC8195007

[31] Tan SS, Saw S, Chew KL, et al. Comparative clinical evaluation of the Roche Elecsys and Abbott severe acute respiratory syndrome coronavirus 2 (SARS-CoV-2) serology assays for coronavirus disease 2019 (COVID-19). Arch Pathol Lab Med. 2021;145(1):32–38. 10.5858/arpa.2020-0499-SA 33367664

[32] Suhandynata RT, Bevins NJ, Tran JT, et al. SARS-CoV-2 serology status detected by commercialized platforms distinguishes previous infection and vaccination adaptive immune responses. J Appl Lab Med. 2021;6(5):1109–1122. 10.1093/jalm/jfab080 34170314 PMC8409063

[33] Stringhini S, Zaballa M-E, Pullen N, et al. Seroprevalence of anti-SARS-CoV-2 antibodies 6 months into the vaccination campaign in Geneva, Switzerland, 1 June to 7 July 2021. Eurosurveillance. 2021;26(43):2100830. 10.2807/1560-7917.ES.2021.26.43.2100830 34713799 PMC8555371

[34] MU Health Care. What you need to know about the Johnson & Johnson COVID-19 vaccine [homepage on the Internet]. 2021 [cited 2022 Jul 10]. Available from: https://www.muhealth.org/our-stories/what-you-need-know-about-johnson-johnson-covid-19-vaccine#:~:text=The scientists behind the Johnson,t replicate or cause illness

[35] The Immunisation Advisory Centre. How cominarty is made and what it contains [homepage on the Internet]. 2022 [cited 2022 July 10]. Available from: https://covid.immune.org.nz/how-comirnaty-made-and-what-it-contains

